# Decreased AMP‐activated protein kinase (AMPK) function and protective effect of metformin in neonatal rat pups exposed to hyperoxia lung injury

**DOI:** 10.14814/phy2.14587

**Published:** 2020-09-22

**Authors:** Abha Yadav, Ujala Rana, Teresa Michalkiewicz, Ru‐Jeng Teng, Girija G. Konduri

**Affiliations:** ^1^ Neonatology Division University of Pittsburgh Medical Center Pinnacle Hospital Harrisburg PA USA; ^2^ Department of Pediatrics Medical College of Wisconsin and Children's Research Institute Children's Wisconsin Milwaukee WI USA

**Keywords:** AMP kinase, angiogenesis, bronchopulmonary dysplasia, prematurity

## Abstract

We investigated the hypothesis that exposure of lungs at the saccular stage of development to hyperoxia leads to persistent growth arrest and dysfunction of 5’AMP‐activated protein kinase (AMPK), a key energy sensor in the cell. We exposed neonatal rat pups from postnatal day 1‐ day 10 (P1–P10) to ≥90% oxygen or control normoxia. Pups were euthanized at P4 or P10 or recovered in normoxia until euthanasia at P21. Half of the pups in each group received AMPK activator, metformin, or saline intraperitoneally from P1 to P10. Lung histology, morphometric analysis, immunofluorescence, and immunoblots were done for changes in lung structure at P10 and P21 and AMPK function at P4, P10, and P21. Phosphorylation of AMPK (p‐AMPK) was decreased in lungs at P10 and P21 in hyperoxia‐exposed pups. Metformin increased the levels of p‐AMPK and PGC‐1α, a downstream AMPK target which regulates mitochondrial biogenesis, at P4, P10, and P21 in hyperoxia pups. Lung ATP levels decreased during hyperoxia and were increased by metformin at P10 and P21. Radial alveolar count and alveolar septal tips were decreased and mean linear intercept increased in hyperoxia‐exposed pups at P10 and the changes persisted at P21; these were improved by metformin. Lung capillary number was decreased in hyperoxia‐exposed pups at P10 and P21 and was restored by metformin. Hyperoxia leads to impaired AMPK function, energy balance and alveolar simplification. The AMPK activator, metformin improves AMPK function and alveolar and vascular growth in this rat pup model of hyperoxia‐induced lung injury.

## Graphical abstract

Hyperoxia‐induced impairment of lung growth in newborn rats is associated with decreased AMP kinase function. Metformin increases AMP Kinase function and restores lung growth in hyperoxia‐exposed neonatal rat pups.

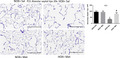

## 1 INTRODUCTION

Bronchopulmonary dysplasia (BPD) is one of the most common morbidities associated with prematurity (Islam, Keller, Aschner, Hartert, & Moore, 2015). Premature infants are born with their lungs at the saccular stage of development and are often exposed to supplemental oxygen for respiratory distress (Higgins et al., 2018). Prolonged exposure of immature lungs to hyperoxia was shown to cause lung injury and impaired growth of the developing lungs (Jobe & Bancalari, 2001). Recent advances in perinatal and neonatal care, including routine use of antenatal steroids and postnatal surfactant led to increased survival of extremely premature infants born at <28 weeks gestational age. These infants born at <28 weeks gestation are at a high risk of developing a new type of BPD, consisting of alveolar simplification with decreases in alveolar septation and vascular growth on lung histology (Abman, Bancalari, & Jobe, 2017). This arrest of lung growth persists even after recovery from respiratory distress and weaning infants off oxygen and respiratory support. The mechanism of initial lung injury that leads to growth arrest is multifactorial and includes an increase in reactive oxygen species, deficiency of antioxidant defenses in premature lung, lung inflammation and loss, or alteration in growth factor levels (Abman et al., 2017). Effective therapies to ameliorate lung injury and to restore lung growth in the affected premature infants are currently not available (Higgins et al., 2018).

Neonatal rodent models have been extensively used for the characterization of lung injury and alveolar and vascular growth impairment after hyperoxia exposure. These models include neonatal rat and mouse pups exposed to variable oxygen concentrations and for variable periods, as reviewed by Berger & Bhandari (2014) and O'Reilly & Thébaud (2014). Rodent pups are born with lungs at the saccular stage of development, similar to premature infants born at 22–32 weeks of gestation (Berger & Bhandari, 2014; O'Reilly & Thébaud, 2014). Rodent pups exposed to hyperoxia environment develop histologic changes similar to BPD and are commonly used to study the pathophysiology of BPD. Previous studies using these models have focused on oxidant‐mediated injury, role of inflammation and cytokines and TGF‐ß signaling pathway (Dasgupta, Sakurai, & Wang, 2009; Datta et al., 2015; Young et al., 2016). There are fewer studies on metabolic alterations in the lung with exposure to hyperoxia and their direct role in the lung growth arrest. Ratner et al reported that neonatal mice exposed to hyperoxia show evidence of mitochondrial injury with Complex‐I dysfunction, decreased ATP levels and cell death in the lungs (Ratner, Starkov, Matsiukevich, Polin, & Ten, 2009).

5’ AMP‐activated protein kinase (AMPK) is a key energy sensor in the cell which maintains cellular homeostasis during stress (Hardie, Ross, & Hawley, 2012). AMPK preserves mitochondrial function during stress through regulation of PGC‐1α, a key transcription factor required for mitochondrial biogenesis (Scarpulla, Vega, & Kelly, 2012). Activation of AMPK is promoted by increased AMP/ATP ratio and phosphorylation at Threonine 172 of the α subunit (α1 or α2) by upstream kinases, liver kinase B1 (LKB1), and CamKinase‐II (Gowans, Hawley, Ross, & Hardie, 2013). Phosphorylated AMPK (p‐AMPK) in turn phosphorylates a number of downstream clients, including PGC‐1α and endothelial nitric oxide synthase (eNOS) (Hardie et al., 2012). The changes in AMPK function and alteration in the regulation of mitochondrial biogenesis by this signaling pathway during hyperoxia remains unclear. We hypothesized that a decrease in AMPK function during hyperoxia leads to decreased PGC‐1α levels and mitochondrial function with depletion of ATP, which contributes to impaired alveolar and vascular growth in the lung. We also hypothesized that the AMPK activator, metformin (Hawley, Gadalla, Olsen, & Hardie, 2002), will promote AMPK function and attenuate the effects of hyperoxia on lung growth. We investigated our hypothesis in a neonatal rat pup model of hyperoxia induced lung injury in the immediate (P4 and P10) and post recovery (P21) periods.

## 2 METHODS

### 2.1 Study protocol

**FIGURE 1:**
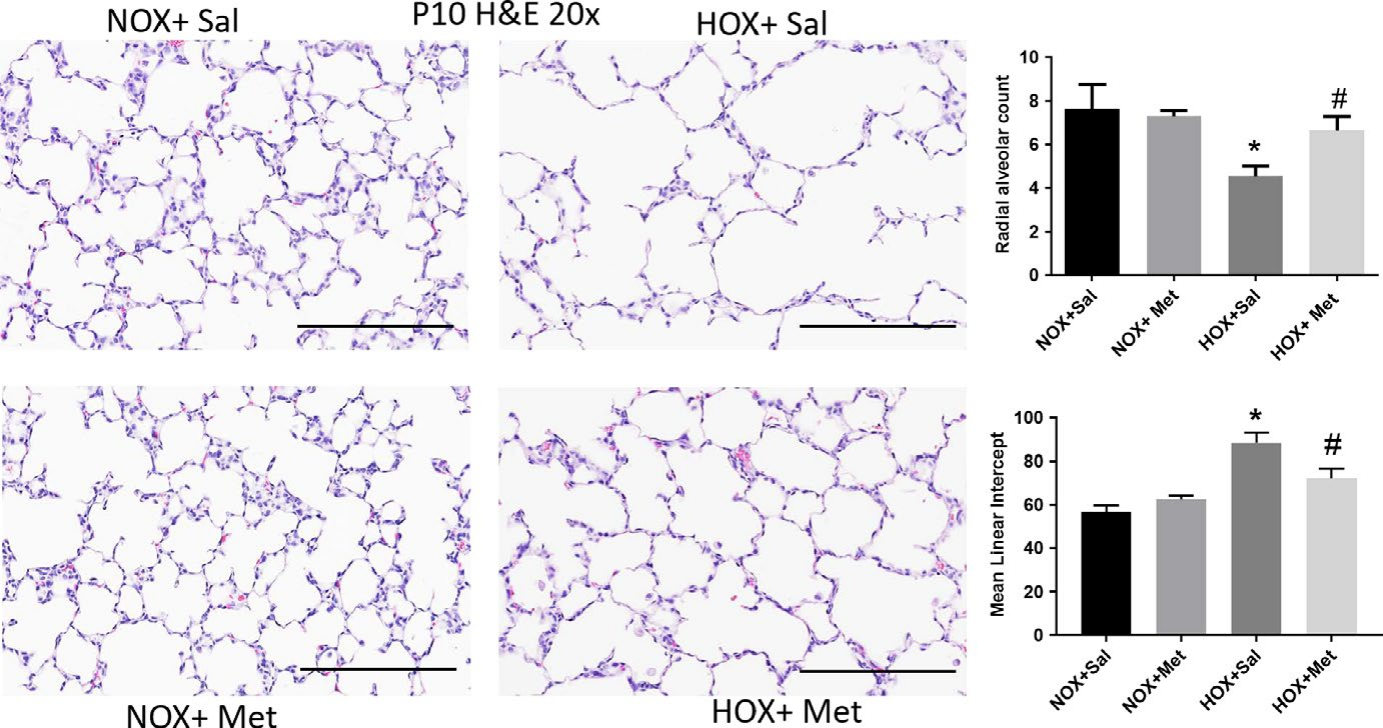
Lung alveolar structure identified by hematoxylin‐eosin (H & E) staining of paraffin embedded lung sections obtained at postnatal day 10 (P10), cut to 5 μm thickness and imaged at 20×. Scale bars are 200 μm. Summary data shown in bar graphs for radial alveolar count (RAC) and mean linear intercept (MLI) were from eight pups in each group and are shown as mean ± *SD*. *Indicates *p* < .05 by ANOVA and Tukey's post‐hoc test from normoxia + saline and # from hyperoxia + saline groups. Hyperoxia decreased the RAC and induced alveolar simplification, indicated by increase in MLI. Metformin increased RAC and decreased MLI in hyperoxia‐exposed pups. HOX, hyperoxia; NOX, normoxia

All animal studies were approved by Medical College of Wisconsin Institutional Animal Care and Use Committee (IACUC) and conformed to the current guidelines of NIH for care and use of laboratory animals (Figure 1). Rats were cared under 12‐hr day/night cycle with unlimited access to chow and water. Sprague‐Dawley rat pups were randomized at birth to either normoxia (21% O_2_) or hyperoxia (≥90% O_2_) and kept with their dam in the chamber at assigned oxygen concentration of 21% or ≥90%. Dams were rotated every 24h between normoxia and hyperoxia to avoid severe lung injury from hyperoxia. Oxygen concentrations were monitored continuously with an oxygen sensor (Drägerwerk AG). Pups in each group were further randomized to receive either metformin 50 mg kg^−1^ d^−1^ or equal volume of normal saline by intraperitoneal (IP) injection until 10 days. This protocol therefore has four study groups and equal numbers of male and female pups were allocated to each of the four study groups. Some pups in each litter randomized to normoxia or hyperoxia were euthanized at postnatal day 4 (P4) to assess AMPK function by western blots on lung lysates. Lung histology was not done at P4 due to small size of the lungs, which precluded adequate and uniform inflation of lungs due to small size of trachea. Some other pups were euthanized at P10 and their lungs were harvested to study the immediate effects of hyperoxia on lung histology and AMPK function. The remaining pups in each group were returned to normoxia and IP injections were stopped. These pups were allowed to recover in 21% O_2_ until P21 and were euthanized to evaluate the persistence of developmental disruption in the lung and AMPK dysfunction after initial hyperoxia injury. Body weights and total lung weights were obtained at the time of each harvest at P4, P10, and P21. Harvested lungs from different pups were inflation fixed at 1.3 kPa (10 cm‐H_2_O) with 10% formalin for lung histology or snap frozen for Western blots and ATP levels. Blood samples were collected at the time of euthanasia at P10 and P21 to test blood glucose levels by glucose oxidase method, using commercially available test strips (Stat Strip, Nova Biomedical). Pups from a minimum of three litters were included in each of the four treatment groups in the study. Samples from at least four pups from different litters were included at P4 and at least eight pups for each variable, including lung morphometry, western blots and ATP levels, at P10 and P21.

### 2.2 Lung histology

Lungs harvested from pups were inflation fixed at 1.3 kPa pressure with 10% formaldehyde infused via the trachea. Lung tissue was then fixed in 10% formalin for 48–72 hr and was subjected to paraffin embedding and sectioning to thickness of 5 microns. The sectioned slides were stained with hematoxylin and eosin and imaged using NanoZoomer 2.0‐HT and NDP scan. Radial alveolar counts (RAC) were obtained using methods we reported previously (Lai et al., 2019). Briefly, to determine the RAC, a line was drawn from the center of the respiratory tract perpendicular to the nearest connective tissue septum on the digital image, and the alveoli intercepting the drawn line were counted. To determine the mean linear intercept (MLI), a horizontal line was drawn on the image and the number of intercepts through the alveolar wall was counted. The MLI was calculated by dividing the length of the line by the sum of the number of traverses. Secondary alveolar septa measurements were also obtained to study lung structure complexity. Lung sections were deparaffinized, rehydrated, and stained with aldehyde fuchsin for elastin. Images of lung specimens were digitally captured under 20× magnification and secondary septa were counted in each image. Lung capillaries were identified in lung sections obtained at P10 by immune histochemistry (IHC) for endothelial cell marker, rat endothelial cell antigen (RECA). IHC was performed using ImmPRESS PLUS Staining Kit (Vector Labs). Briefly, the tissue sections were deparaffinized, rehydrated, and treated with antigen retrieval buffer (pH 6.0) (DAKO) for 20 min at 95°C. The endogenous peroxidase activity was blocked using the Bloxall solution. After blocking serum treatment, tissue sections were incubated with RECA‐1 (Abcam) antibody overnight. The following day, lung sections were incubated with anti‐mouse ImmPress polymer reagent. Images were developed using chromogen (ImmPACT DAB EqV) for <2 min and were counterstained with hematoxylin, followed by mounting on glass slides.

Immunofluorescence (IF) with CD31 antibody was used to identify capillaries in P21 lung sections, as the larger size of the pups allowed us to clear the blood from the lungs effectively using HBSS to perfuse lungs from right ventricle, to avoid non‐specific staining for CD31 from retained blood cells. For these studies, deparaffinized lung sections were rehydrated and treated with antigen retrieval solution as mentioned above. The sections were blocked and incubated with CD31 antibody (Monoclonal mouse anti‐CD31 Ab, Thermo Fisher Scientific) overnight, followed by secondary antibody (Alexa Fluor 488, Thermo Fisher Scientific) incubation next day. The sections were mounted on glass slides using Progold mounting medium with DAPI (Thermo Fisher Scientific). The stained tissue sections were imaged at 40× on a confocal microscope at 488 nm (green) and 405 nm for DAPI.

### 2.3 Western blots

For immunoblotting, the following reagents were used: Rabbit Anti‐phos‐^Thr172^AMPK‐α1 polyclonal Ab and mouse Anti‐p‐^Ser1177^eNOS Monoclonal Ab were from Cell Signaling Technology. Mouse anti PGC1α monoclonal Ab was from Millipore Sigma. Mouse anti‐β‐actin Ab (1:10,000) was obtained from Santa Cruz Biotechnology. Lung samples were lysed in MOPS (3‐[*N*‐morpholino] propane sulfonic acid) buffer. The cell lysate was sonicated, and cell debris was removed by centrifugation at 9600 *g*. Protein content of the lysate was determined by bicinchoninic acid method. Proteins were separated by SDS‐PAGE, transferred to nitrocellulose membranes, and were blotted with antibodies for Phos‐^Thr172^AMPKα1, PGC‐1α, p‐eNOS, and β‐actin overnight at 4°C. The membranes were blotted with horseradish peroxidase‐conjugated anti‐mouse or anti‐rabbit IgG antibody (1:9,000; Bio‐Rad) and exposed to CL‐XPosure films (Pierce) after treatment with SuperSignal West Pico (Pierce). The signals were analyzed with ImageJ and normalized to the expression of β‐actin as loading control.

### 2.4 Measurement of ATP levels

ATP levels were measured using a bioluminescence assay kit (Millipore Sigma, www.sigmaaldrich.com) following the manufacturer's instructions. Samples from P10 and P21 lungs were lysed in the lysis buffer supplied in the kit and ATP levels were determined by Firefly luciferase bioluminescence method, using a multi‐mode plate reader (Perkin Elmer). Standard curves were run for each assay and concentration of ATP in the sample was obtained from the standard curve. Assays were run in triplicate for each sample. Protein concentration of each sample lysate was measured and ATP concentration in each sample was normalized to protein concentration of the sample and expressed in picomoles/mg protein.

### 2.5 Statistical analysis of data

Data are shown as mean ± *SD*. Data were analyzed by two‐way ANOVA, accounting for exposure to normoxia or hyperoxia and to metformin or saline. When significant differences (*p* < .05) were found, Tukey's post hoc test was done to determine which groups were different.

## 3 RESULTS

### 3.1 Effects of hyperoxia and metformin on alveolar growth in newborn rat pups

**FIGURE 2:**
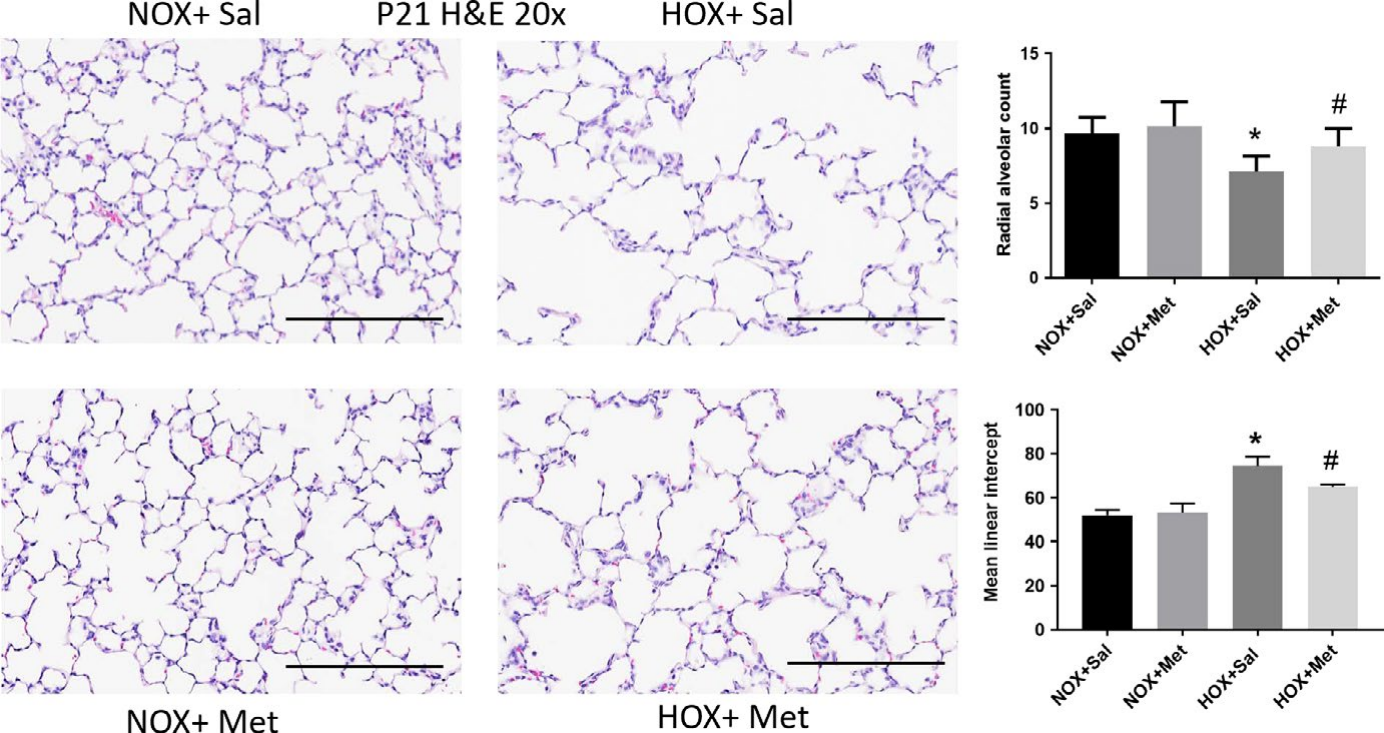
Lung alveolar structure identified by hematoxylin‐eosin (H & E) staining of paraffin embedded lung sections obtained at postnatal day 21 (P21), cut to 5 μm thickness and imaged at 20×. Scale bars are 200 μm. Summary data shown in bar graphs for radial alveolar count (RAC) and mean linear intercept (MLI) were from eight pups in each group and are shown as mean ± *SD*. * Indicates *p* < .05 by ANOVA and Tukey's post‐hoc test from normoxia + saline and # from hyperoxia + saline groups. Pups exposed to hyperoxia from P1 to P10 continue to show decreased RAC and alveolar simplification at P21 despite 10 days of recovery in normoxia. Metformin treatment from P1 to P10 increased RAC and improved alveolar simplification at P21, indicated by decreased MLI compared to saline‐treated hyperoxia pups. HOX, hyperoxia; NOX, normoxia

**FIGURE 3:**
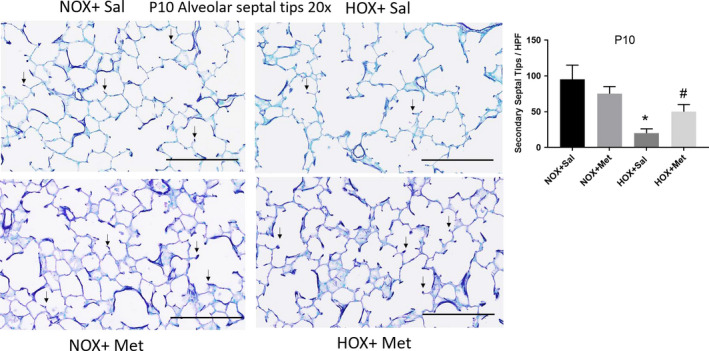
Alveolar septal tips identified by aldehyde fuchsin staining of paraffin embedded lung sections obtained at postnatal day 10 (P10), cut to 5 μm thickness and imaged at 20×. Scale bars are 200 μm. Summary data shown in bar graphs are for number of secondary septal tips per high power field, indicated by small black arrows. *N* = 8 pups per group and data are mean ± *SD*. * Indicates *p* < .05 by ANOVA and Tukey's post‐hoc test from normoxia + saline and # from hyperoxia + saline groups. Hyperoxia decreased the number of secondary septal tips at P10. Pups treated with metformin during hyperoxia exposure had increased number of septal tips, suggesting improved alveolarization in this group. HOX, hyperoxia; NOX, normoxia

**FIGURE 4:**
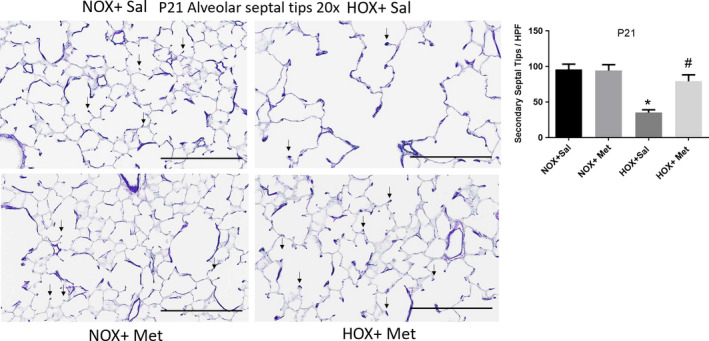
Alveolar septal tips identified by aldehyde fuchsin staining of paraffin embedded lung sections obtained at postnatal day 21 (P21), cut to 5 μm thickness and imaged at 20×. Scale bars are 200 μm. Summary data shown in bar graphs are for the number of secondary septal tips per high power field, indicated by small black arrows. *N* = 8 pups per group and data are mean ± *SD*. * Indicates *p* < .05 by ANOVA and Tukey's post‐hoc test from normoxia + saline and # from hyperoxia + saline groups. Pups exposed to hyperoxia from P1 to P10 continue to show a decrease in the number of secondary septal tips at P21, despite normoxia recovery. Hyperoxia pups treated with Metformin from P1 to P10 continue to show increased number of septal tips compared to hyperoxia + saline, suggesting improved alveolarization at P21. HOX, hyperoxia; NOX, normoxia

Lung histology demonstrated decreased alveolar number and simplification with enlarged airspaces at P10, at the end of hyperoxia exposure (Figure 1). These changes persisted to P21, despite recovery of the hyperoxia‐exposed pups in normoxic environment for 10 days (Figure 2). These changes were associated with a significant decrease in radial alveolar count (RAC) and increase in MLI, which corresponds to enlarged airspaces (Figures 1 and 2). Consistent with alveolar simplification, hyperoxia decreased the number of secondary alveolar septal tips, visible with aldehyde Fuchsin staining for elastin, both at P10 (Figure 3) and P21 (Figure 4). The changes were not different between male and female pups, so the data for both sexes were combined into one group. Pups treated with metformin showed no changes during normoxia. However, metformin increased the RAC and decreased MLI in hyperoxia‐exposed pups, compared to saline‐treated pups (Figure 1). The alveolar structure revealed less simplification in the metformin treated, hyperoxia‐exposed pups at P10 (Figure 1). The improvement persisted at P21, after discontinuation of metformin at the end of hyperoxia exposure at P10 (Figure 2). Metformin‐treated pups also had increased alveolar septation, indicated by increased number of secondary alveolar septal tips in the hyperoxia‐exposed pups at both P10 and P21 (Figures 3 and 4). These data demonstrate that metformin treatment during hyperoxia exposure protects the lung from growth arrest and alveolar simplification.

### 3.2 Effects of hyperoxia and metformin on capillary density in the lung

**FIGURE 5:**
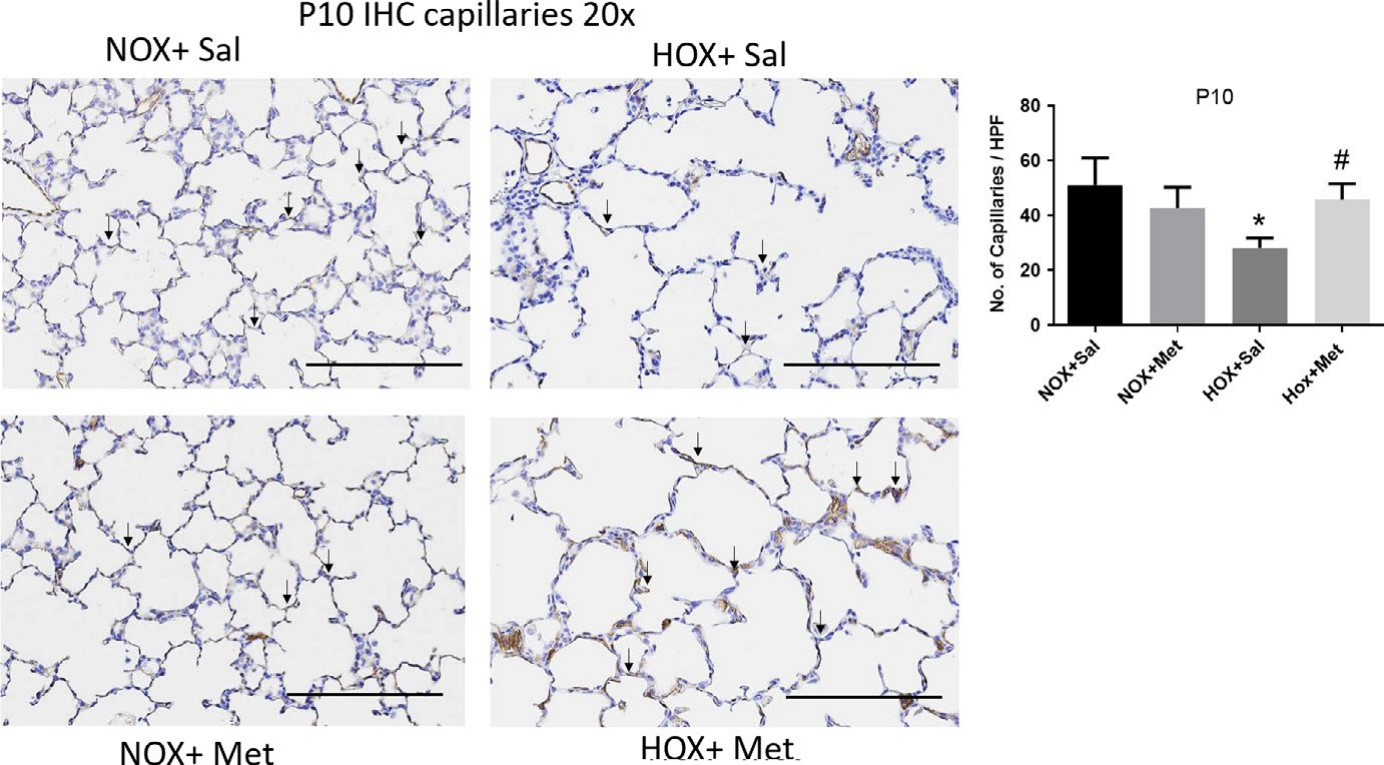
Capillaries in lung sections identified by immunohistochemistry (IHC) staining for rat endothelial cell antigen and imaged at 20× in P10 rat pups. Scale bars are 200 μm. Capillaries identified by brown staining are indicated by black arrows in representative lung sections from the four groups of rat pups. Summary data shown in bar graphs are for the number of capillaries per high power field, displayed as mean ± *SD* for *n* = 8 in each group. * Indicates *p* < .05 by ANOVA and Tukey's post‐hoc test from normoxia + saline and # from hyperoxia + saline groups. Hyperoxia decreased the number of capillaries per high power field in the lung. Hyperoxia pups treated with metformin show higher capillary number per high power field at P10 compared to hyperoxia + saline group. HOX, hyperoxia; NOX, normoxia

**FIGURE 6:**
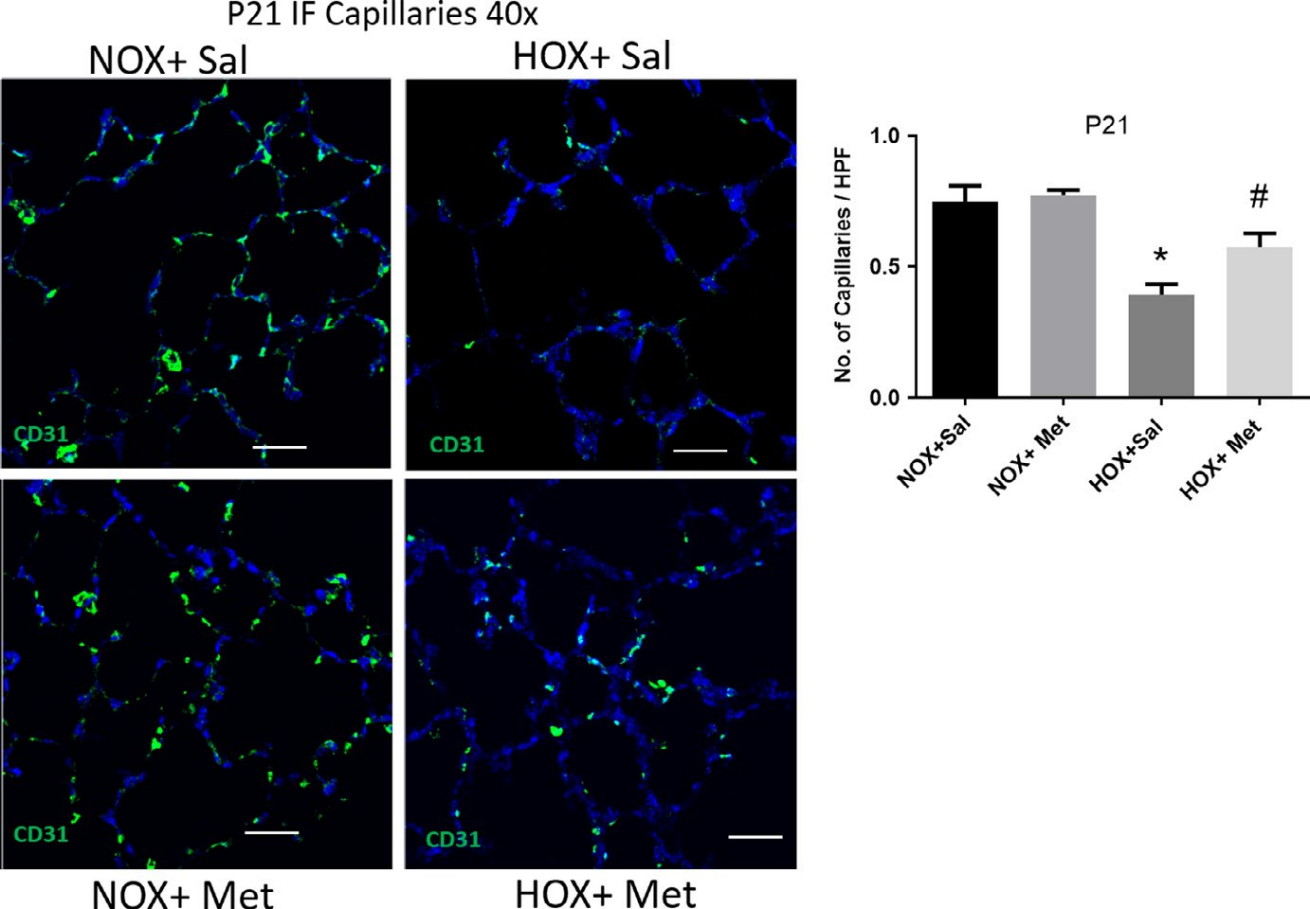
Capillaries in lung sections from P21 rat pups, identified by immunofluorescence (IF) staining for CD31 antigen and detected by Alexa Fluor 488 nm (green) secondary in endothelial cells and imaged at 40×. Scale bars are 50 μm. DAPI staining (blue) was applied to outline the alveolar structure in the background. Summary graph to the right shows the number of capillaries per high power field, displayed as mean ± *SD* for *n* = 8 in each group. * Indicates *p* < .05 by ANOVA and Tukey's post‐hoc test from normoxia + saline and # from hyperoxia + saline groups. Capillaries identified by green staining show persistent decrease in hyperoxia‐exposed pup lungs, despite return to normoxia for the preceding 10 days. Hyperoxia pups treated with metformin continue to show higher number of capillaries per high power field at P21 compared to saline‐treated hyperoxia‐exposed pups. HOX, hyperoxia; NOX, normoxia

Hyperoxia significantly decreased the capillary number in the lungs, as seen by RECA IHC staining in P10 lung sections (Figure 5). The decrease in capillary number persisted to P21, as seen by CD31 IF staining in P21 lung sections (Figure 6). Treatment of rat pups with metformin for 10 days during hyperoxia exposure restored the capillary number both at P10 (Figure 5) and at P21 (Figure 6). These data show that metformin protects lung vascular growth during hyperoxia exposure, and this effect is persistent even after discontinuation of hyperoxia and metformin.

### 3.3 Metformin improves AMPK function in hyperoxia‐exposed pups

**FIGURE 7:**
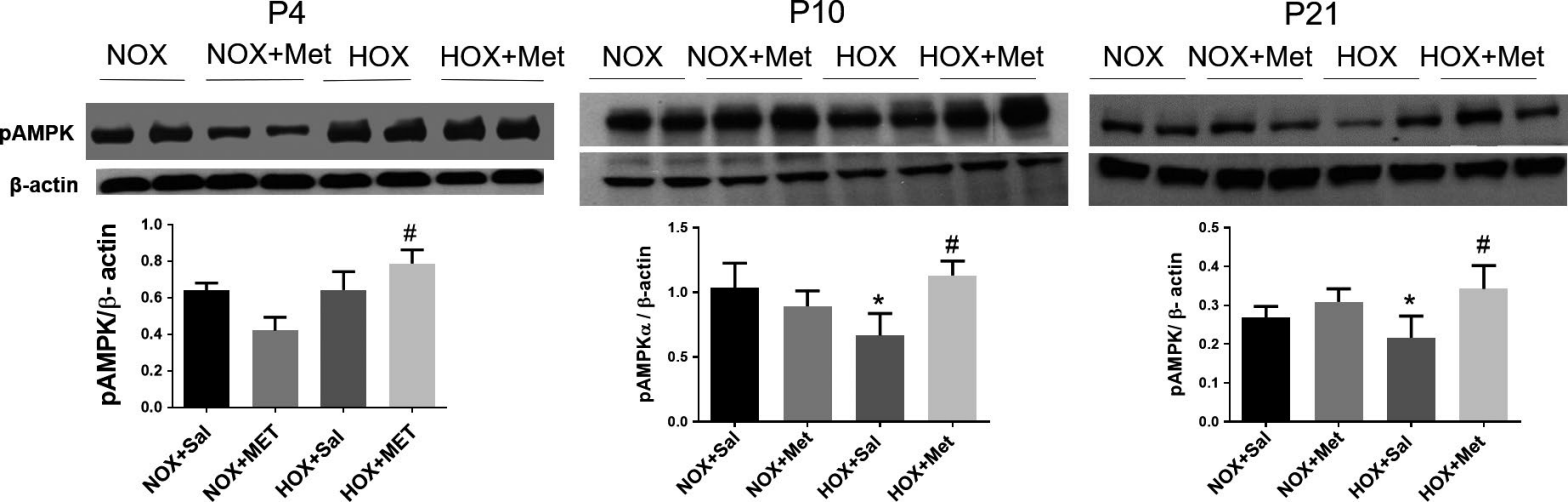
Representative immunoblots for phos‐^Thr172^AMPK‐α1 from the lung homogenates obtained for the four groups of rat pups at postnatal day 4 (P4), P10 and P21. Summary data are shown in bar graphs as mean ± *SD* for *n* = 4 at P4 and *n* = 8 at P10 and P21. * Indicates *p* < .05 by ANOVA and Tukey's post‐hoc test from normoxia + saline and # from hyperoxia + saline groups. Hyperoxia decreased the p‐AMPK protein level at P10 and P21 lung samples. P‐AMPK protein levels increased in metformin‐treated hyperoxia group at all three ages, P4, P10, and P21. HOX, hyperoxia; NOX, normoxia

**FIGURE 8:**
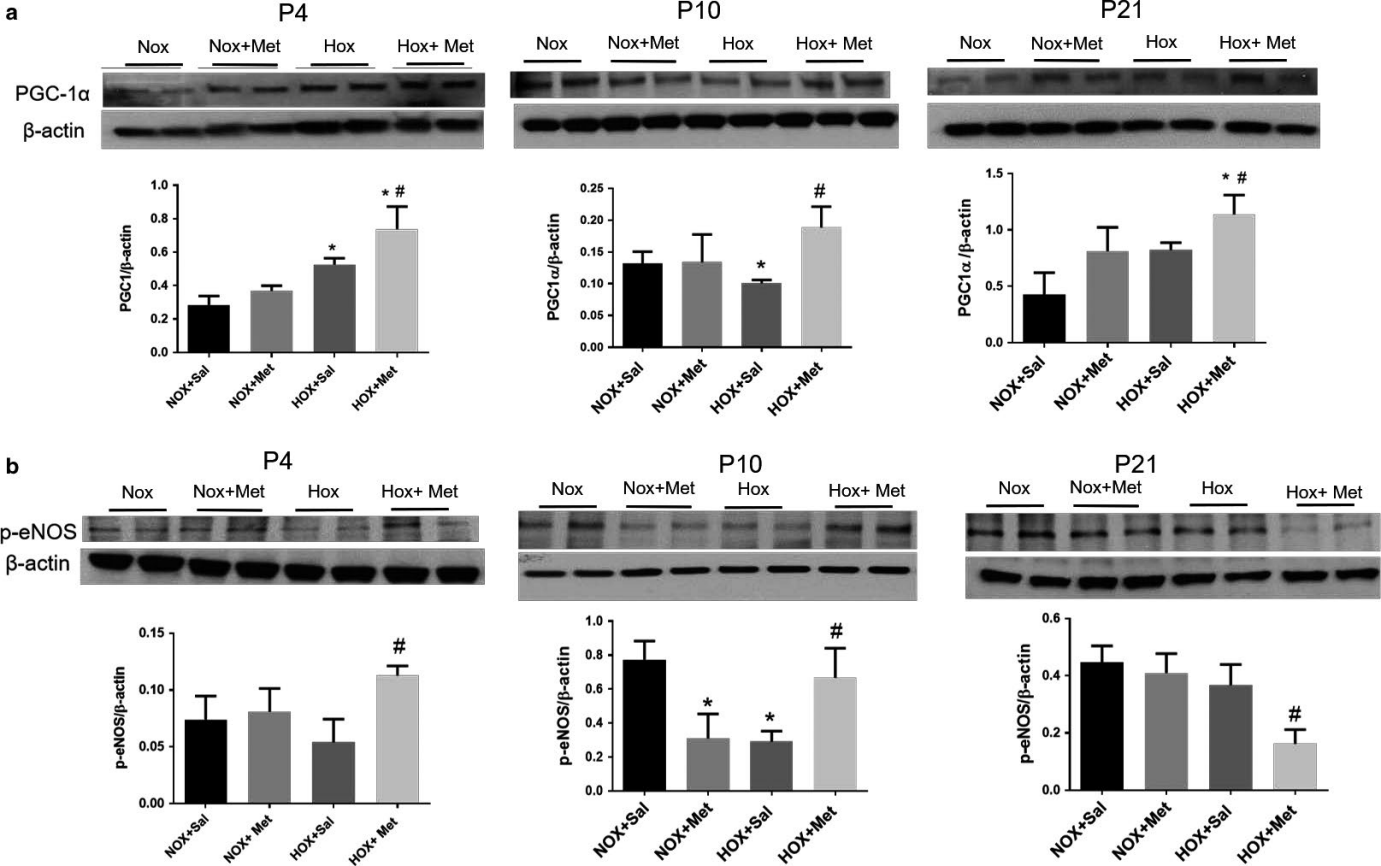
Representative immunoblots and summary data for PGC‐1α (a) and p‐^Ser1177^eNOS (b) in lung samples from the four groups of rat pups at postnatal day 4 (P4), P10 and P21. Summary data are shown in bar graphs as mean ± *SD* for *n* = 4 at P4 and *n* = 8 at P10 and P21. * Indicates *p* < .05 by ANOVA and Tukey's post‐hoc test from normoxia + saline and # from hyperoxia + saline groups. Hyperoxia increased the PGC‐1α protein level at P4 and decreased it in P10 lung samples. PGC‐1α protein levels increased in metformin‐treated hyperoxia group at all three ages, P4, P10, and P21. Hyperoxia has decreased the p‐eNOS levels at P10. Metformin increased p‐eNOS levels at P4 and P10 during hyperoxia. The p‐eNOS levels decreased at P21 in metformin‐treated hyperoxia‐exposed pups compared to saline‐treated pups. HOX, hyperoxia; NOX, normoxia

The level of Thr172 phosphorylated AMPK, the functionally active form of AMPK, did not change at P4 in the hyperoxia‐exposed rat pups treated with saline and was increased in hyperoxia pups treated with metformin (Figure 7). In contrast, p‐AMPK levels decreased in saline treated hyperoxia‐exposed pups at P10, compared to normoxia‐exposed pups (Figure 7). The decrease in p‐AMPK levels persisted at P21, in saline‐treated hyperoxia‐exposed pups, despite recovery in normoxia for 10 days (Figure 7). Treatment of pups in hyperoxia with metformin increased p‐AMPK levels at P10 (Figure 7). The higher levels of p‐AMPK persisted at P21 in the metformin‐treated hyperoxia group, despite discontinuation of hyperoxia and metformin at P10 (Figure 7). These data suggest that AMPK phosphorylation is maintained early during hyperoxia exposure up to 4 days, followed by a decline at 10 days of hyperoxia and persistent decline at P21, despite recovery in normoxia. Metformin increased the phosphorylation of AMPK consistently during and after hyperoxia exposure. Consistent with improved AMPK function, the levels of PGC‐1α, a key transcription co‐factor involved in mitochondrial biogenesis, increased in pups that received metformin during hyperoxia exposure, compared to saline treated hyperoxia pups (Figure 8). PGC‐1α levels increased in hyperoxia + saline group at P4, with further increase in hyperoxia + metformin group (Figure 8). PGC‐1α levels decreased significantly in hyperoxia + saline group at P10 and were increased by metformin in the hyperoxia group. Metformin treated hyperoxia pups had persistent increase in PGC‐1α levels at P21, despite discontinuation of metformin and hyperoxia at P10 (Figure 8). We also observed that ^Ser1177^phosphorylation of eNOS, another target of AMPK, was increased at P4 and P10, in the metformin‐treated hyperoxia‐exposed pups (Figure 8). Phos‐eNOS levels decreased in metformin treated normoxia pups at P10 and metformin treated hyperoxia pups at P21 (Figure 8). These data suggest that increased phosphorylation of eNOS by AMPK occurs only during hyperoxia + metformin treatment and the increase does not persist after discontinuation of metformin. These data also suggest that phosphorylation of eNOS is independent of the AMPK effects on PGC‐1α. The reason for decreased p‐eNOS levels observed in metformin‐treated normoxia pups at P10 remains unclear.

### 3.4 Metformin improves ATP levels in hyperoxia‐exposed pups

**FIGURE 9:**
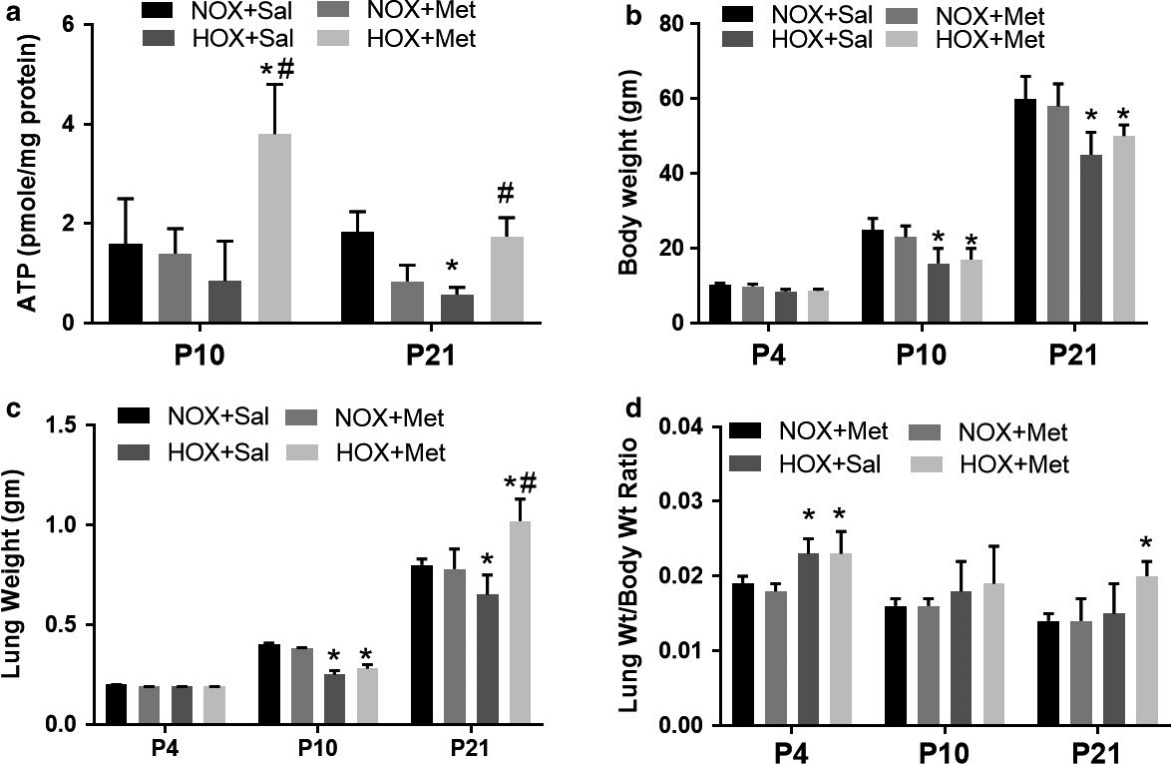
Effect of hyperoxia and metformin on lung ATP levels, body weight, and lung weights in neonatal rat pups. Data are mean ± *SD* for *n* = 4 at P4 and *n* = 8 at P10 and P21. * Indicates *p* < .05 by ANOVA and Tukey's post‐hoc test from normoxia + saline and # from hyperoxia + saline groups. (a) Hyperoxia decreased the ATP levels significantly at P21 from normoxia + saline group. Metformin treatment of hyperoxia‐exposed pups increased the ATP levels at both P10 and P21. (b) Body weights were not different at P4 but were significantly lower in both hyperoxia‐exposed groups at P10 and P21. (c) Lung weights were significantly lower in hyperoxia + saline group at P10 and P21. Metformin‐treated hyperoxia pups had higher lung weights at P21. (d) Lung weight to body weight ratio was higher at P4 in both hyperoxia‐exposed groups and was not different at P10 or P21 in the hyperoxia + saline group. Lung weight to body weight ratio was higher in metformin‐treated hyperoxia pups at P21. HOX, hyperoxia; NOX, normoxia

Lung ATP levels were lower after hyperoxia exposure at P10; however, the difference was not statistically significant (*p* = .08) (Figure 9). The lung ATP levels were significantly lower in hyperoxia‐exposed pups at P21 despite 10 days of normoxia recovery, suggesting persistent mitochondrial dysfunction and altered energy balance after neonatal hyperoxia exposure (Figure 9). Metformin treatment increased the ATP levels in hyperoxia‐exposed pups both at P10 and P21, compared to saline‐treated hyperoxia‐exposed pups (Figure 9). These data are consistent with increased AMPK function and higher PGC‐1α levels in response to metformin treatment in the hyperoxia‐exposed pups.

### 3.5 Effects of hyperoxia and metformin on lung and body weights of pups

Body weights of the pups treated during normoxia with metformin were not different from saline‐treated normoxia pups (Figure 9). Hyperoxia decreased the pup body weight at P10 and this weight difference persisted at P21 in both saline and metformin treated groups (Figure 9), despite normoxia recovery. Metformin‐treated hyperoxia pups had similar body weight to saline treated pups at both P10 and P21. The lung weights also showed a decrease in saline‐treated hyperoxia‐exposed pups at P10 and P21 (Figure 9). Metformin‐treated hyperoxia pups showed higher lung weight at P21. Lung weight to body weight ratio was higher early in hyperoxia‐exposed groups at P4, compared to normoxia‐exposed group. Whether this is related to lung edema from injury early in exposure to hyperoxia is not clear from our study. The ratio was not different in the hyperoxia‐exposed pups compared to normoxia pups at P10 (Figure 9). However, metformin‐treated hyperoxia pups had increased lung weight to body weight ratio at P21 compared to saline‐treated pups (Figure 9).

### 3.6 Effect of metformin on blood glucose levels

The blood glucose levels checked on the pups at P4, P10, and P21 were not different in the metformin treated pups from saline‐treated pups in either normoxia or hyperoxia groups (data not shown). The glucose levels ranged from 100 to 150 mg/dl with no differences between any of the four groups at the three postnatal ages evaluated.

## 4 Discussion

Premature infants born at 22–32 weeks of gestation have lungs at the saccular stage of development; they are vulnerable to injury from therapies used to support their respiratory function (Abman et al., 2017). Supplemental oxygen is one of the most common therapies used to maintain physiological levels of oxygenation in premature neonates. However, supplemental oxygen can injure the lungs and interrupt normal alveolar and vascular growth. This growth arrest is the hallmark of “new BPD” which occurs more commonly in extremely premature infants born at <28 weeks gestation in the current era of neonatal intensive care (Abman et al., 2017). The mechanisms of lung growth arrest after recovery from hyperoxia remain unclear. Our studies provide evidence that hyperoxia decreases the function of AMPK, a key sensor of cellular energy balance. Our novel studies show that activation of AMPK with metformin, improves lung growth in hyperoxia‐exposed newborn rat pups. Our studies suggest that AMPK activation is a novel therapeutic target to improve lung energy balance and to restore lung growth when the use of supplemental oxygen becomes necessary in premature infants.

Previous studies have extensively investigated the role of oxidative stress, inflammation, and activation of epithelial mesenchymal transition as a basis for lung injury and altered trajectory of lung development in response to hyperoxia (Buczynski, Maduekwe, & O'Reilly, 2013; Dasgupta et al., 2009; Datta et al., 2015; Morty, 2018; Teng et al., 2017). Whether hyperoxia leads to altered mitochondrial function and energy balance in the cells is less clear. Ratner et al reported that mouse pups exposed to hyperoxia develop complex‐I dysfunction and energy failure (Ratner et al., 2009). A decrease in mitochondrial function and mitochondrial DNA variation were linked to hyperoxia‐induced lung growth arrest in neonatal mice (Kandasamy, Rezonzew, Jilling, Ballinger, & Ambalavanan, 2019) and in premature infants with BPD (Kandasamy, Olave, Ballinger, & Ambalavanan, 2017). Since cell replication and differentiation are energy‐dependent processes, it is likely that mitochondrial dysfunction underlies the lung growth arrest, which involves both alveolar and vascular compartments of the lung.

AMPK is a key energy sensor in the cell and initiates downstream signaling to maintain energy balance and cellular homeostasis in response to stress. AMPK is activated when AMP/ADP levels increase with depletion of ATP (Gowans et al., 2013). AMPK is a heterotrimeric protein consisting of α, β, and γ subunits. When AMP binds the γ subunit, the resulting conformational change allows ^Thr^172 on the α subunit to become accessible to phosphorylation by upstream kinases, LKB1, and Cam Kinase‐II. Phosphorylation of AMPKα activates AMPK, which in turn phosphorylates a number of downstream targets, such as eNOS, a key enzyme involved in lung angiogenesis and growth (Balasubramaniam, Maxey, Morgan, Markham, & Abman, 2006; Hardie et al., 2012; Teng et al., 2013). These diverse effects help orchestrate adaptive mechanisms that promote cell survival by turning off key energy consuming pathways, such as protein synthesis and promote efficient energy production by maintaining the mitochondrial function. We observed that AMPK function as assessed by ^Thr^172 phosphorylation, was maintained early during hyperoxia, but declines after 10 days of hyperoxia. It is unclear whether the later decline in function is related to decrease in upstream kinases or adaptation to ongoing injury and persistence of ROS with hyperoxia exposure longer than 3 days. Previous studies in cultured human lung fibroblasts exposed to 70% O_2_ for 24 hr reported increased AMPK function (Klimova et al., 2009). We did not assess AMPK phosphorylation in the lung at such an early stage of hyperoxia exposure. However, we observed that p‐AMPK levels after 4 days of hyperoxia exposure were preserved, with a decline occurring after 10 days of hyperoxia. These results from two studies likely represent time dependent changes in the response of AMPK to hyperoxia exposure, with early activation followed by decline with continued exposure.

One of the important downstream targets of AMPK is PGC‐1α, a nodal point in mitochondrial biogenesis, which is required for continuous supply of ATP and metabolic intermediates needed for cell proliferation (Liang & Ward, 2006). AMPK regulates PGC‐1α expression and its function through phosphorylation. We found that there is a coordinated downregulation of p‐AMPK and PGC‐1α levels in the lung after 10 days of hyperoxia exposure in the rat pups. We also observed persistence of these changes at P21, despite recovery in normoxia for an additional 10 days. These data suggest that AMPK dysfunction persists during recovery and can lead to sustained alterations in energy metabolism in the lung as a basis for growth arrest. Consistent with these observations, we found a decrease in lung ATP levels at P21 in the hyperoxia‐exposed lungs. ATP levels were restored by metformin administration during hyperoxia, suggesting that decrease in AMPK function may be linked to decrease in ATP levels during hyperoxia exposure. Previous studies demonstrated that AMPK regulates mitochondrial function and energy balance through maintaining levels of PGC‐1α, a key transcription co‐factor required for mitochondrial biogenesis (Scarpulla et al., 2012). We recently reported that AMPK function regulates PGC‐1α levels and mitochondrial biogenesis, in the pulmonary artery endothelial cells, using a genetic loss and gain of function approach (Rana et al., 2020). Our data overall suggest that altered energy balance is a component of lung injury after exposure of neonatal lung to hyperoxia. We investigated changes in eNOS phosphorylation since eNOS function is required for lung development (Balasubramaniam et al., 2006) and is facilitated by AMPK. Our data suggest that AMPK activation by metformin promotes eNOS phosphorylation, which was maintained through the period of alveolarization at P10. Whether this effect of metformin is required to promote lung growth is not clear from our study. However, eNOS phosphorylation was not maintained after discontinuation of metformin. Endothelial NOS is also phosphorylated by Akt and other upstream kinases (Fulton et al., 1999). Whether alterations in these pathways contribute to decrease in eNOS phosphorylation during recovery is unclear from our studies.

We found that activation of AMPK with metformin increases p‐AMPK and PGC‐1α levels in the lung at all three phases of hyperoxia exposure and recovery. Consistent with these changes, ATP levels were higher at P10 and P21, suggesting improved energy balance with metformin administration in the hyperoxia‐exposed lungs. These changes were associated with significant increase in RAC and elastin positive alveolar septal tips and a corresponding decrease in MLI. These data demonstrate improved alveolarization in rat pups treated with metformin during hyperoxia. Additionally, we observed an increase in capillary number in the hyperoxia lungs when pups were treated with metformin. Levels of p‐^Ser^1177‐eNOS, a key driver of angiogenesis, were higher at P4 and P10, during hyperoxia and metformin treatment. Phosphorylation at ^Ser^1177 site was known to activate eNOS to increase NO synthesis (Fulton et al., 1999). Whether activation of eNOS contributes to improved vascular growth with metformin treatment is not clear from our studies. In contrast to these changes, metformin decreased p‐AMPK levels at P4 in normoxia lungs and p‐eNOS levels at P10 in normoxia lungs. The mechanism for decreased activation of AMPK and eNOS in normoxia lungs is unclear from our studies. Metformin is known to have AMPK independent effects, primarily through inhibition of mitochondrial complex‐I function, which can increase or decrease reactive oxygen species, based on the dose of metformin and redox state of the cell (Vial, Detaille, & Guigas, 2019). Whether a difference in redox state in the lung led to divergent results during normoxia and hyperoxia requires further investigation. Metformin increased the lung weights and lung weight to body weight ratio at P21 after hyperoxia exposure. Whether this results from better alveolar and vascular growth is also unclear from our study, since increase in lung edema, which would be an adverse effect of metformin, could also result in similar increase in lung weight. However, a previous study in neonatal rat pup model of hyperoxia injury demonstrated that metformin decreases inflammation in the lung (Chen et al., 2015). Our studies overall suggest that AMPK dysfunction in the lungs contributes to impaired lung growth from hyperoxia exposure. We observed that lung weight to body weight ratio was higher at P4 in both hyperoxia groups. A previous study of sequential changes in lung injury and inflammation observed that lung edema increases to maximal level at 3 days and resolves at days 6 and 9 during hyperoxia exposure in neonatal rat pups (Ben‐Ari et al., 2000). These data are consistent with lung edema as a potential mechanism for the relative increase in lung weight at P4. However, we did not assess for lung edema in our studies.

Neonatal rodent models have been extensively used to study the altered lung biology observed in premature infants. Rat and mouse pups are born with lungs at the saccular stage, which extends from embryonic day 18.5 (E18.5) to P4 (Berger & Bhandari, 2014; O'Reilly & Thébaud, 2014). Premature infants born at 22–32 weeks of gestation also have lung development at the saccular stage and are at the highest risk of BPD in response to hyperoxia or mechanical ventilation (Abman et al., 2017). Alveolar stage of development extends from P4‐P10 in the rodent pups and alveolarization is completed by P21 (O'Reilly & Thébaud, 2014). These developmental stages guided our use of hyperoxia exposure from P1 to P10 and examination of lungs both at P10 and P21 in our rat pups. The persistent growth arrest we observed at P21 simulates the persistent alveolar simplification observed in extremely premature infants that develop BPD. Our results suggest that AMPK dysfunction contributes to persistent decrease in septation and capillary development in this model. Whether improved lung growth with metformin persists into adult life requires further investigation.

Previous studies investigated the effects of AMPK activation with metformin on the hyperoxia‐induced lung injury and in pulmonary hypertension. Chen et al. (2015) reported that metformin decreases lung inflammation in rat pups exposed to 100% O_2_ for 10 days (P1–P10) when it was given in doses of 25–100 mg kg ^−1^d^−1^. They reported decreases in alveolar wall thickness and infiltration of macrophages and neutrophils in the lungs of hyperoxia‐exposed pups. Metformin did not improve alveolar or vascular growth or right ventricular hypertrophy in this study. The study did not address changes in AMPK phosphorylation or expression of PGC‐1α. Our study used 50 mg kg^−1^ d^−1^ dose of metformin, as a midpoint between the doses used in the study by Chen et al. Our study also differs from the study by Chen et al in using ~90% O_2_ exposure from P1 to P10 and assessed the effects of metformin on both immediate (P10) and post normoxia recovery (P21) stages of hyperoxia exposure. We found improvement in lung growth and vessel number with the conditions we used for our study. Endothelial AMPK has been previously shown to protect from pulmonary hypertension in adult rats (Omura et al., 2016). Metformin has also been tested previously in pulmonary hypertension models in neonatal and adult animals. Metformin decreased the pulmonary artery pressure and medial thickening in both hypoxia and monocrotaline induced pulmonary hypertension in adult rats (Li et al., 2016). Metformin attenuated the increase in right ventricular pressure and medial thickening of pulmonary arteries, increased the lung vessel density and improved alveolar simplification in a neonatal lamb model of persistent pulmonary hypertension (Rana et al., 2020). Our present study did not investigate the effects of metformin on pulmonary hypertension; however, we observed higher capillary number in the lungs in hyperoxia‐exposed pups. We also did not investigate the changes in inflammation in response to hyperoxia or metformin in the current studies.

Our study has some limitations that we wish to acknowledge. Although we did not observe sex specific differences in the effects of hyperoxia or metformin on the lung alveolar or capillary count, our sample size was small and a larger number of pups may reveal such differences as observed previously with hyperoxia in mouse pups (Lingappan et al., 2013). We did not investigate whether changes in cell apoptosis and proliferation occur in the lung during hyperoxia and in response to AMPK activation. We also did not study other AMPK downstream mechanisms that contribute to improved lung growth with metformin. We measured the effects of metformin on blood glucose only at the end of 4 and 10 days periods of administration and not during the administration of daily doses. Any potential decreases in blood glucose earlier in the time course of metformin administration would not be revealed in our study. It is also unclear whether the increase in lung weight to body weight ratio we observed after metformin administration during hyperoxia was due to lung growth or increase in lung water content. Although term rat pups have lungs at the saccular stage similar to premature infants, they do not share the structural immaturity of the lungs and decreased maturity of the other organs that may contribute to lung growth arrest in premature infants. We also observed that pups subjected to 90% O_2_ for 10 days had smaller weights, compared to their normoxia counterparts. Postnatal growth restriction also has been shown to decrease lung growth (Wedgwood et al., 2016). Whether impaired alveolar development in our study resulted from hyperoxia alone or from additional effects of growth restriction is unknown from our study. Hyperoxia‐exposed pups also did not regain their weight to the level of control, normoxia‐exposed pups at P21, despite being in normoxia from P10 to P21. The mechanism for the persistence of growth impairment in the hyperoxia group is unclear from our studies. Our study also did not address whether catch up growth may occur beyond P21, since the study period ended at P21. We observed that lung weight to body weight ratio did not change in hyperoxia. The dose of metformin we used is also at the higher end of therapeutic doses used in adults with type2 diabetes and metabolic syndrome. However, we did not observe changes in glucose levels at the end of 10‐day course of metformin.

In conclusion, our study provides evidence that AMPK dysfunction contributes to impaired lung growth in the neonatal rat pup model of hyperoxia‐induced lung growth arrest. We found that the AMPK agonist, metformin, improved AMPK function and lung growth in this model. Whether this signaling pathway is a potential therapeutic target for premature infants with BPD requires additional investigation.

## CONFLICTS OF INTEREST

None of the authors have financial conflicts of interest for the work presented in this manuscript.

## AUTHOR CONTRIBUTIONS

**AY** conducted hyperoxia studies, western blots and ATP assays and prepared the initial draft of the manuscript. **UR** performed the lung histology imaging and IHC and Immunofluorescent imaging in lung sections. **TM** performed the lung harvest, lung inflations and hyperoxia studies. **RT** performed western blots and edited the manuscript and **GGK** conceived the study, designed the study protocol, oversaw western blots, assays and imaging studies, analyzed the data, prepared the final draft of the manuscript and proofed the manuscript and figures for submission.
